# Quercetin protects rat cortical neurons against traumatic brain injury

**DOI:** 10.3892/mmr.2022.12732

**Published:** 2022-05-10

**Authors:** Guoliang Du, Zongmao Zhao, Yonghan Chen, Zonghao Li, Yaohui Tian, Zhifeng Liu, Bin Liu, Jianqiang Song

Mol Med Rep 17: 7859–7865, 2018; DOI: 10.3892/mmr.2018.8801

Subsequently to the publication of the above paper, an interested reader drew to the authors’ attention that a pair of the data panels in Fig. 4A (on p. 7862), showing the ‘Sham’ and ‘TBI’ experiments, were overlapping, such that the data were apparently derived from the same original source.

After having examined their original data, the authors have realized that they uploaded the incorrect image for the ‘Sham’ experiment in this figure. The revised version of Fig. 4 showing the correct data for all the experiments portrayed in Fig. 4A, is shown opposite. Note that the replacement of the erroneous data does not affect either the results or the conclusions reported in this paper, and all the authors agree to the publication of this corrigendum. The authors are grateful to the Editor of *Molecular Medicine Reports* for granting them this opportunity to publish a Corrigendum, and apologize to the readership for any inconvenience caused.

## Figures and Tables

**Figure 4.**
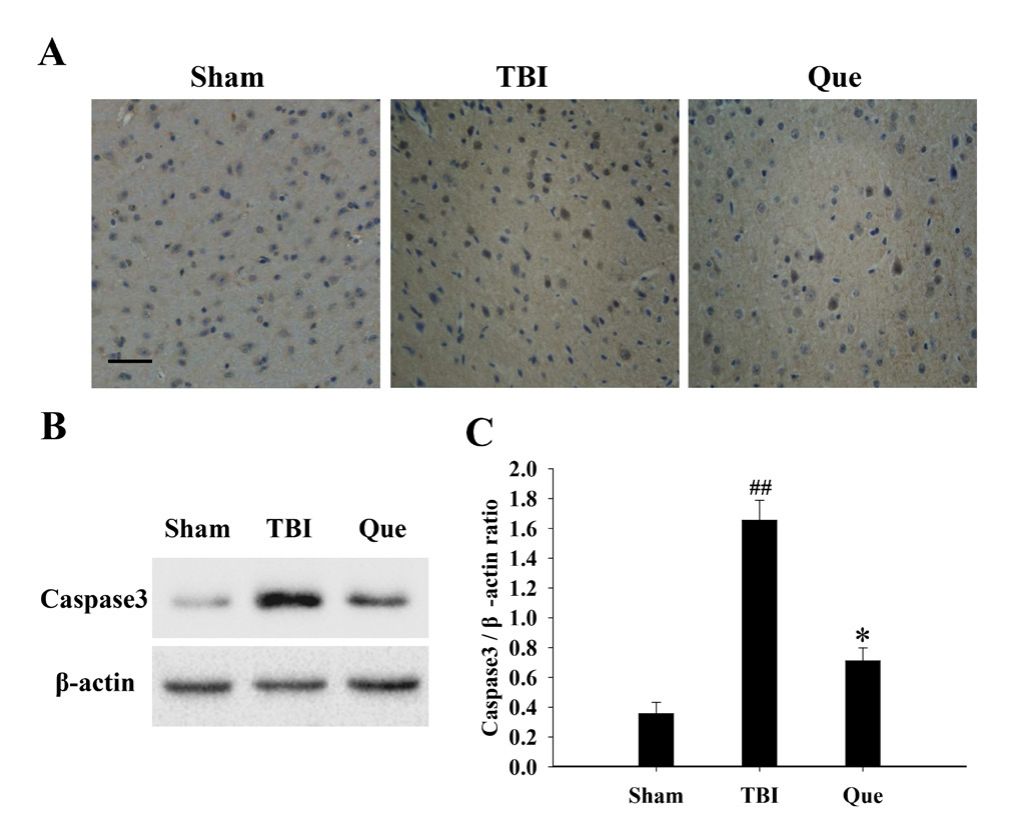
Effect of quercetin on Caspase3 expression following TBI. (A) Representative images of immunohistochemical analysis of Caspase3 in the sham, TBI and Que groups at 24 h. Scale bar, 100 µm. (B) Western blot analysis demonstrating expression levels of caspase3 in the cortex of rats at 24 h. (C) The quantitative results of caspase3 are expressed as the ratio of densitometries of caspase3 to β-actin bands. Bars represent the mean ± standard error of the mean (n=5/group). The results demonstrated that the protein levels of caspase3 increased significantly in the TBI group at 24 h (^##^P<0.01 vs. sham group), and the levels of caspase3 exhibited significant downregulation at following treatment with quercetin (*P<0.05 vs. TBI group). TBI, traumatic brain injury; Que, quercetin-treated.

